# Knowledge, Attitude, and Practice Associated with Antibiotic Use among University Students: A Survey in Nepal

**DOI:** 10.3390/ijerph16203996

**Published:** 2019-10-18

**Authors:** Poonam Shah, Rajeev Shrestha, Zongfu Mao, Yilin Chen, Yan Chen, Pramesh Koju, Xinliang Liu, Hao Li

**Affiliations:** 1 School of Health Sciences/Global Health Institute, Wuhan University, Wuhan 430072, China; shpoonam77@gmail.com (P.S.); zfmao@126.com (Z.M.); ychen725@whu.edu.cn (Y.C.); xlliu921006@gmail.com (X.L.); 2 Pharmacovigilance Unit, Dhulikhel Hospital, Kathmandu University Hospital, Dhulikhel 45200, Nepal; rmaleku@hotmail.com (R.S.); kojupramesh@gmail.com (P.K.); 3 Department of Pharmacology, Kathmandu University School of Medical Sciences, Dhulikhel 45200, Nepal; 4 Department of Global Health, University of Washington, Seattle, WA 98105, USA; ylcheneve@gmail.com; 5 School of Health Sciences, Faculty of Biology, Medicine and Health, The University of Manchester, Manchester M13 9PL, UK

**Keywords:** antibiotic use, attitude, knowledge, practice, university students, Nepal

## Abstract

The purpose of this study was to conduct a preliminary study to assess knowledge, attitude, and practice (KAP) associated with antibiotic use among medical students (MS) and non-medical students (NMS) at Kathmandu University, Nepal. A self-administered questionnaire was distributed to 1223 students for a cross-sectional study. In total, 1222 questionnaires collected from 609 MS and 613 NMS were regarded as effective. A *t*-test and Chi-square test were applied to analyze the data. A total of 25 out of 39 questions in the KAP survey were found to have statistical significance. The MS showed higher levels of knowledge/attitude/practice associated with antibiotic use than the NMS. Significant gaps were found in and between the MS and NMS in the first and final years of study. Interventions, such as lectures, courses, workshops, and seminars on antibiotic use, along with internet and media campaigns, etc., are needed to improve the awareness and change the behavior of both the MS and the NMS of universities with regards to the rational use of antibiotics.

## 1. Introduction

Antimicrobial resistance (AMR) is currently a hot debate. Its hazards have been underestimated in low- and middle-income countries (LMICs) [1,2]. The World Health Organization (WHO) recognizes AMR as a major global health problem that threatens our ability to treat diseases and requires urgent action [3]. The overuse, underuse, and misuse of antibiotics result in antimicrobial resistance problems worldwide [4].

A review on antimicrobial resistance produced in 2014 estimated that the year’s annual mortality attributable to AMR was 700,000, and that the number may rise to 10 million by 2050 if no actions are taken to reduce the inappropriate use of antibiotics [5]. In various studies, it has been found that taking an inappropriate dosage of antibiotics can result in the development of resistant bacteria and diminish the ability of the oral flora to resist the colonization of harmful micro-organisms, thereby leading to super infections caused by multi-resistant bacteria [6,7]. In particular, second- or even third-line antibiotics may be required, which are very expensive, and lead to prolonged hospitalization and more side effects [8,9,10].

Moreover, the misuse of antibiotics in treating viral infections is common and the prevalence of self-medication is alarmingly high in developing countries [2,3,4]. Studies in Palestine, Jordan, China, and India found that the irrational use of antibiotics among students due to a lack of knowledge, attitude, and practice has deteriorated antibiotic resistance [11,12,13,14]. Nepal has been listed as a less developing country and is far behind the comprehensive governance of AMR. Antibiotics can be sold by unqualified personnel. People can easily buy antibiotics at any clinics, without a prescription. A lack of effective regulatory bodies and operative control mechanisms has caused the irrational and inappropriate use of antibiotics in Nepal [15].

There is limited evidence regarding antibiotic resistance in Nepal. Recent studies of acute respiratory infections have indicated that more than half the cases analyzed were resistant to first-line antibiotics [16]. The National Antimicrobial Containment Action Plan Nepal 2016 was released based on WHO guidelines, and was designed to guide actions in a common effort to address urgent and serious drug-resistant threats that affect people and to take urgent action for combating resistance at national, regional, and local levels [17]. In this plan, arousing the awareness of the inappropriate use of antibiotics has been set as one of the priority areas in combating antibiotic resistance, while only a few studies on this topic have been conducted in Nepal. Nayak et al. highlighted the need for further studies among health professionals to strengthen the curriculum on antibiotics use and self-medication practices in Nepal [18]. Less concerns are focused on medical students (MS) and non-medical students (NMS), despite the fact that their knowledge, attitude, and behavior regarding the use of antibiotics have a tremendous impact on the consequences associated with antibiotic use. Only a few studies have been conducted so far among nursing and dental background students in Nepal [18,19]. The purpose of this study was to assess the knowledge, attitude, and practice (KAP) associated with antibiotics use among medical and non-medical students at Kathmandu University, Nepal.

## 2. Methods

### 2.1. Study Setting and Design

The research protocol was approved by the Institutional Review Committee School of Medical Science/Dhulikhel Hospital (IRC-KUSMS), with the approval number 129/17. A cross-sectional survey was designed to be carried out at Kathmandu University. The minimum sample size was calculated based on the formula below:

(1) $$\mathrm{Sample}\;\mathrm{Size}\;=\frac{\frac{z^{2}\times p\left(1-p\right)}{e^{2}}}{1+\frac{z^{2}\times p\left(1-p\right)}{e^{2}N}}$$

where *N* = 15,200 (population size); *e* = 0.05 (margin of error); *z* = 1.96 (confidence level); and p = 0.05.

Based on the above parameters, the estimated minimum sample size was 375. However, in order to improve the reliability of the data, the sample was enlarged to 1223 students. The MS students included undergraduate students in nursing, dentistry, medicine and surgery (MBBS). Certificate-level students and pharmacy students were excluded from the MS because they already have a professional knowledge of antibiotics. The NMS were from computer engineering and mechanical engineering, working towards a Bachelor of Technology degree in environmental science, computer science, electrical and electronic engineering, civil engineering, and chemical science.

### 2.2. Survey Tool Development, Pre-Testing, and Validation

The questionnaire was developed based on previous studies carried out in China, India, and Saudi [13,20,21]. It was piloted among 10 bachelor students. The questionnaire was further revised based on their feedback and further advice from two senior experts. The final questionnaire was comprised of four categories and included 44 questions on the following: (1) demographics characteristics (5 questions); (2) knowledge of antibiotics (15 questions); (3) attitude towards antibiotic use in terms of the severity of antibiotic abuse, its influence on students, and reasons for abuse (12 questions); and (4) practice with regards to antibiotic use (12 questions related to sources of getting antibiotics knowledge, sources of information, eagerness to learn related knowledge, college curriculum arrangement, and the appropriate use of antibiotics).

### 2.3. Data Collection

The survey was carried out from 10 December 2017 to 28 February 2018. Two research investigators were trained and they distributed the self-administered questionnaires to students in the classrooms after obtaining verbal approval from the school departments. Participation in the survey was fully voluntary and written consent was obtained from each of the participants. The objectives of the study, confidentiality of individual information, and other ethical considerations mentioned in the survey guidelines were explained to the participants prior to data collection. They were asked to answer as many of the questions as they could. However, if they were not sure about the answer, they could just leave it blank. Altogether, 1223 questionnaires were collected anonymously without the presence of the investigators.

### 2.4. Grading Method

A common grading method was used for each item in the KAP questionnaire. One point was given to the correct option, and zero to the wrong/unanswered option. Multiple answers to each of the correct options were given one score; otherwise, no score was given. The final scores were summarized for each of the individuals.

### 2.5. Data Processing and Analysis

The data was entered into EpiData Entry for data documentation. SPSS was applied to further analyze the data. One participant was excluded from the sample because all the answers given were the same. Variation in some of the questions was observed. Descriptive statistics were employed to summarize the data. After the scored data was checked for a normal distribution, no violations were observed. *T*-tests were applied to compare mean values of the variables represented by questions. A Chi-square test was used for a comparative analysis of categorical variables. *p* < 0.05 was regarded as statistically significant. In our early stage analysis, significant differences between the first and final year students were found, so a more in-depth comparative analysis of the two groups was conducted. Moreover, in our analysis, only those questions with statistical significance between the MS and NMS were presented and discussed. For the convenience of our discussion, these questions have been relabeled.

## 3. Results

### 3.1. Demographic Characteristics of the Sample

The effective response rate of the sample was 99.1%. Of the 1222 respondents, 609 were MS and 613 were NMS from different programs. The demographic background of the subjects varied: among the 609 MS, 222 (36.3%) were male and 387 (63.7%) were female, whereas among the 611 NMS, 526 (85.8%) were male and only 87 (14.2%) were female. Table 1 shows the detailed demographic characteristics of the students from different programs by number and percentage, including the gender, years of study, average monthly family income, and places to get medical care.

### 3.2. Knowledge Level of Antibiotic Use by the MS and NMS

The overall scores for the MS were significantly higher than those of the NMS in terms of their knowledge about antibiotic use (mean: 10.98 vs. 8.60 (*p* < 0.001)). Table 2 shows the knowledge level of the MS and NMS for nine questions. In general, for each of the questions, the percentage of the MS who gave a right answer was significantly higher than that of the NMS. Special attention needs to be given to questions K3, K5, and K8, as both the MS and NMS had a low percentage of giving the right answer. Similar findings were found for the first and last year students, respectively.

The knowledge level between first-year and last-year students can be further described in the two groups. For the MS, the first year students showed a lower knowledge level than the last year students for questions K1–K4 and K6–K9, while the former showed an even higher knowledge level than the latter for question K5. For the NMS, the first-year students had a lower knowledge level than the final-year students for questions K2, A3, and K7–K9, while the former showed a higher knowledge level than the final-year students for K1, K4, K5, and K6.

### 3.3. Attitude Level of Antibiotic Use by the MS and NMS

Our results indicated that the MS had a higher score for the level of attitude towards antibiotic use than the NMS (mean: 6.894 vs. 6.0458, respectively; *p* < 0.001). Table 3 shows the level of attitude of antibiotic use by the MS and NMS for eight questions. For each of the questions, the MS had a higher percentage than that of the NMS. In particular, for questions A2, 3, and 4, the percentages of the NMS were much lower than those of the MS. For Questions A5 and A8, both the MS and the NMS had a very low percentage. Similar findings can be observed for the first-year and last-year students, respectively.

The attitude level of first-year and last-year students can be further described in two groups. For the MS, the first-year students showed a lower attitude level than the last-year students for questions A1–A5, A7, and A8, while the former showed an even higher attitude level than the latter for question A5. For the NMS, the first-year students had a lower attitude level than the final-year students for questions A2–A8, while the former showed a higher attitude level than the final-year students for A1.

### 3.4. Practice Level of Antibiotic Use by the MS and the NMS

Overall, the MS scored higher than the NMS in terms of the practice of antibiotic use (mean: 12.9 vs. 11.7, respectively; *p* < 0.001). Table 4 displays the practice level of antibiotic use by the MS and NMS for eight questions. In general, the percentage of MS was higher than NMS in terms of giving the right answers. Special attention needs to be paid to questions P2 and P4. Both the MS and NMS had a low percentage of giving the right answers. Similar findings can be found in the first- and final-year students, respectively.

The practice level of the first-year and last-year students can be further described in two groups. For the MS, the first-year students showed a lower practice level than the last-year students for questions P1, P3, P5, P6, and P8, while the former showed an even higher practice level than the latter for questions P2, P4, and P7. For the NMS, the final-year students only showed a higher practice level for question P4, while for the other questions (P1–P3 and P5–P8), the first-year students showed a higher practice level than the final-year students.

## 4. Discussion

In our study, we found that the MS showed higher levels of knowledge/attitude/practice associated with antibiotic use than the NMS in general. Most of our findings are consistent with other studies in Nepal and other countries [12,13,14,16,18,19]. However, for some of the KAP questions, both the MS and NMS exhibited a low level, with the NMS displaying a lower level than the MS. Like similar research, these findings not only indicated the necessity of launching specific interventions for both groups respectively and collectively, but also identified the priority areas for improvement [20].

### 4.1. Knowledge Regarding Antibiotic Use

In our knowledge survey part, only 54% of the students knew that antibiotics should not be used to cure infections caused by viruses (K3), only 53.5% of the students knew that a chest infection should not always be treated with antibiotics (K5), and only 31.6% of the students thought that antibiotics could not speed up the recovery process of a cold and cough (K8). For the three questions, the students, either classified by MS and NMS, or by the first-year and last-year students, all showed a low level of knowledge, which may impact their attitude toward antibiotic use. This also indicated that, even though, for most of the questions, the MS did better than the NMS, there is still space to increase the knowledge level of the MS. Moreover, for most of the questions, it is easy to infer that the final-year MS showed a higher knowledge level than the first-year MS, indicating the effectiveness of the medical courses in terms of increasing the knowledge of antibiotic use. For the NMS, five out of nine questions were even answered better by the first-year students than the last-year students, which may reflect the situation that there is a lack of systematic interventions for their knowledge on antibiotic use in the university among the NMS.

Our findings call for interventions targeting the two groups respectively and collectively. It is recommended that a medication course is opened and handheld devices of medication are applied for the MS [21], highlighting knowledge on the rational use of antibiotics [22]. More activities can be organized for both the MS and NMS, such as lectures by doctors and pharmacists, workshops, seminars, contests on knowledge of antibiotic use, broadcasts, etc. Nowadays, antibiotic resistance has aroused many concerns and debates in the TV, press, and Internet, etc., which raised the public awareness of its hazards [7], while in our findings, the third most preferred way (after the hospital and clinic) to get information on antibiotics was via the media. It is therefore recommended that media approaches, such as TV programs, printed/internet news, radio, etc., could be effective ways of disseminating knowledge on the rational use of antibiotics to all university students.

### 4.2. Attitude Regarding Antibiotic Use

In our attitude survey part, a majority of the MS showed a high attitude level for questions A1–A3, A6, and A7, indicating a positive impact of knowledge on attitude. However, an improvement in knowledge may not necessarily lead to a higher level of attitude, which is supported by our findings for questions A4, A5, and A8, in which the MS showed a very low level of attitude. Question A4 reflected that more NMS than MS were not aware of the harm of antibiotic resistance. Questions A5 and A8 can show that the MS already had a good knowledge about antibiotic use and many of them were quite confident about the rational use of antibiotics along with saving time and money [19]. Moreover, for questions A5 and A8, both the MS and NMS had a low attitude level, indicating the room for improvement from knowledge to attitude.

Our findings call for more interventions to make students’ attitude in line with their knowledge regarding antibiotic use. In particular, more focus should be put on the NMS, as they showed a much lower attitude level than the MS for most of the questions. Apart from actions to increase their knowledge on campus as a way to increase their attitude and from public media, doctors and pharmacists could be motivated to inform students about knowledge on antibiotic use. For the MS, their attitude can be further improved through internships, along with courses and public media. In particular, they can learn more from doctors and pharmacists about rational medication. However, doctors and nurses need to be educated as well in order to make sure that they can have a more positive impact on the attitude of the MS.

### 4.3. Practice Regarding Antibiotic Use

In our practice survey part, the MS showed a higher practice level for all the questions in general, indicating a higher level of medical knowledge on better practice regarding antibiotic use. However, it can also be found that the percentage of giving the right answers was low for questions P2 and P4, indicating that improvement in knowledge and attitude may not necessarily lead to a higher level of practice. It is interesting to see that, except for P4, the NMS students in the final year showed a lower practice level of antibiotic use than the first year, which may be explained by the fact that with more exposure to antibiotics, the former exhibited more misuse of antibiotics due to a lack of enough knowledge on antibiotic use.

Our findings call for tailored interventions to be provided to university students to improve their practice level. Internships in hospitals could be a good way for the MS to improve both their attitude level and practice level whilst supervised by their doctors, provided that the doctors conform to guidelines on rational prescription and medication in practice [18]. Relevant laws, regulations, guidelines, etc. regarding the rational use of prescriptions and selling of antibiotics need to be formulated by governments and hospitals, which can restrict the access to the unconditional purchasing of antibiotics by patients and their relatives (including students) from hospitals and clinics [23,24]. Besides, not only the MS, but also the NMS, can learn from doctors and pharmacists on the proper use of antibiotics when they are sick and communicate with their doctors and pharmacists.

### 4.4. Strengths and Limitations of the Study

This study has a big sample size compared with two similar studies conducted in Nepal among 176 nursing and dental students [18], and among 330 nursing students [19]. Most of the questions we listed in Table 2, Table 3 and Table 4 should be given special attention. From our findings, it is easy to view the priorities for the university with regards to developing interventions for the MS and NMS respectively and collectively. Our study also has some limitations. One is related to the data processing. Although we eliminated one invalid questionnaire from the study, we were unable to differentiate those blank answers that were not filled in by the participants due to being forgotten about from those that the participants did not know how to answer. Another is that it was only conducted at just one university and it has not yet been scaled up to the KAP survey conducted at other universities in order to understand the whole situation of KAP among university students in Nepal.

## 5. Conclusions

Our study found a significant gap in the level of knowledge/attitude/practice of antibiotic use between the MS and NMS, and between the first- and last-year study students. Education on the rational use of antibiotics should be strengthened through/by classes, workshops, seminars, etc. at the university, along with public approaches, such as the Internet, media, etc. It is necessary to establish a specific course on the rational use of medications that highlights the questions we identified in our KAP survey. The findings of this survey can be disseminated among the students to make them better understand their knowledge, attitude, and practice on antibiotic use. More research is needed regarding how to integrate better knowledge into attitude and further into practice.

## Figures and Tables

**Table 1:** Characteristics of 1222 Subjects.

Demographic Characteristics	Medical Students (MS) n (%)	Non-Medical Students (NMS) n (%)
**Gender**		
Male	222 (36.3)	526 (85.8)
Female	387 (63.7)	87 (14.2)
**Years of Study**		
First Year	179 (29.6)	274 (44.7)
Second Year	152 (25.2)	150 (24.5)
Third Year	125 (20.7)	102 (16.6)
Fourth Year	148 (24.5)	87 (14.2)
**Family income per month on average (NRs)**		
<20,000	47 (7.9)	95 (16.3)
20,000–30,000	131 (22.0)	152 (26.1)
30,000–40,000	161 (27.1)	146 (25.1)
>40,000	256 (43.0)	189 (32.5)
**Places to get medical care**		
Hospital	497 (81.6)	413 (67.8)
Clinic	94 (15.4)	126 (20.7)
Medicine Shops	11 (1.8)	49 (8.0)
Ayurvedic	4 (0.7)	12 (2.0)
Others	3 (0.5)	9 (1.5)
Total	609 (49.83)	613 (50.16)

**Table 2:** Priority items of students’ knowledge about antibiotic use.

Questions (Correct Answer)	Whole %(n)					First Year %(n)				Final Year %(n)			
	Total %(n)	Medical Students (MS)	Non-Medical Students (NMS)	X^2^	*p*	MS	NMS	X^2^	*p*	MS	NMS	X^2^	*p*
K1. Are there any bacteria in human bodies which can be helpful for us? (Yes)	95.5 (1168/1218)	99.0 (602/608)	92.8 (566/610)	30.4	<0.001	98.9 (176/178)	91.9 (250/272)	10.9	0.004	100 (148/148)	89.7 (78/87)	15.92	<0.001
K2. Can antibiotics be used to cure infections caused by bacteria? (Yes)	86.9 (1057/1216)	96.2 (585/608)	77.6 (472/608)	93.9	<0.001	91.1 (163/179)	73.1 (198/271)	24.2	<0.001	99.3 (147/148)	80.5 (70/87)	27.72	<0.001
K3. Can antibiotics be used to cure infections caused by viruses? (No)	54.0 (656/1215)	73.7 (449/609)	34.2 (207/609)	199.6	<0.001	69.3 (124/179)	32.1 (87/271)	62.9	<0.001	76.4 (113/148)	39.5 (34/86)	36.26	<0.001
K4. Is it okay to stop taking antibiotics without finishing complete dose if you are feeling well? (No)	85.2 (1039/1220)	94.1 (573/609)	76.3 (466/611)	78.82	<0.001	90.5 (162179)	78.4 (214/273)	13.6	0.001	98.6 (146/148)	77.0 (67/87)	30.59	<0.001
K5. Should chest infection always be treated with antibiotics? (No)	53.5 (430/1216)	57.9 (351/606)	49.2 (300/610)	106.9	0.001	57.0 (102/179)	50.9 (139/273)	13.6	0.001	53.4 (78/146)	47.1 (41/87)	53.21	<0.001
K6. Can antibiotics cause any side effects? (Yes)	74.8 (903/1208)	87.7 (529/603)	61.8 (374/605)	108.9	<0.001	78.2 (136/174)	65.2 (176/270)	10.8	0.005	95.3 (141/148)	50.6 (44/87)	66.16	<0.001
K7. Have you heard the term “Antibiotic Resistance”? (Yes)	73.9 (901/1219)	97.7 (594/605)	50.2 (307/606)	355.8	<0.001	95.5 (171/179)	44.1 (120/272)	124.6	<0.001	98.6 (145/147)	62.1 (54/87)	57.464	<0.001
K8. Can antibiotics speed up the recovery process of cold and cough? (No)	31.6 (384)	39.4 (239)	23.8 (145)	43.87	<0.001	42.4 (75)	22.4 (61)	20.25	<0.001	46.6 (69)	28.7 (25)	12.43	0.002
K9. Do you think frequent use of antibiotics can decrease occurrence of infection? (No)	79.0 (957)	85.5 (519)	72.2 (438)	45.28	<0.001	76.8 (136)	70.5 (191)	7.191	0.027	87.2 (129)	74.1 (63)	11.108	0.004

*p* < 0.05, statistically significant.

**Table 3:** Priority items of students’ attitude towards antibiotic use.

Questions (Correct Answer)	Whole %(n)					First Year %(n)				Final Year %(n)			
	Total %(n)	Medical Students (MS)	Non-Medical Students (NMS)	X^2^	*p*	MS	NMS	X^2^	*p*	MS	NMS	X^2^	*p*
A1. Do you think there exists misuse of the antibiotics? (Yes)	92.0 (1123/1220)	96.0 (586/609)	87.9 (537/611)	31.7	<0.001	95.5 (171/179)	91.2 (249/273)	3.10	0.212	97.3 (144/148)	86.2 (75/87)	11.0	0.004
A2. Is antibiotic resistance a problem in Nepal? (Yes)	62.2 (755/1214)	82.9 (501/604)	41.6 (254/610)	22.5	<0.001	77.1 (135/175)	41.6 (114/274)	56.1	<0.001	86.5 (128/148)	44.2 (38/86)	48.4	<0.001
A3. Is the overuse of antibiotics result antibiotic resistance? (Yes)	68.2 (830/1217)	90.6 (549/606)	46.0 (281/611)	282.1	<0.001	78.1 (139/178)	42.3 (116/274)	56.8	<0.001	96.6 (143/148)	62.1 (54/87)	50.8	<0.001
A4. Does antibiotic resistance affect you or your family’s health? (Yes)	58.9 (715/1213)	75.8 (458/604)	42.2 (257/609)	193.9	<0.001	69.5 (123/115)	42.3 (115/272)	35.0	<0.001	79.1 (117/148)	52.9 (46/87)	37.7	<0.001
A5. Would you prefer a prescription containing antibiotics during chest infection? (No)	28.6 (345/1205)	21.7 (131/604)	35.6 (214/601)	28.7	<0.001	32.4 (57/176)	35.8 (97/271)	0.6	0.459	16.2 (24/148)	36.6 (30/82)	12.2	<0.001
A6. Do you think that taking less antibiotics than prescribed is more beneficial? (No)	83.2 (1009/1213)	90.6 (552/609)	75.7 (457/604)	48.7	<0.001	88.3 (158/179)	77.9 (211/271)	7.9	0.005	94.6 (140/148)	81.2 (69/85)	10.5	0.001
A7. Is it necessary to know “Rational use of antibiotics”? (Yes)	97.5 (1186/1216)	99.7 (607/608)	95.2 (579/608)	26.8	<0.001	100 (179/179)	95.6 (260/272)	0.0	0.004	100 (148/148)	96.5 (83/86)	5.2	0.022
A8. Would you visit for follow-up after taking antibiotics? (Yes)	23.7 (289/1218)	19.0 (116/609)	28.4 (173/609)	14.74	<0.001	24.0 (43/179)	22.1 (60/272)	0.236	0.627	14.9 (22/148)	41.9 (36/86)	21.3	<0.001

*p* < 0.05, statistically significant.

**Table 4:** Priority items of students’ practice on antibiotic use.

Questions (Correct Answer)	Whole %(n)					First Year %(n)				Final Year %(n)			
	Total %(n)	Medical Students (MS)	Non-Medical Students (NMS)	X^2^	*p*	MS	NMS	X^2^	*p*	MS	NMS	X^2^	*p*
P1. Do you consult a doctor before starting an antibiotic? (Yes)	89.3 (1089/1220)	92.3 (562/609)	86.3 (527/611)	11.6	0.001	88.8 (159/179)	89.8 (246/274)	0.104	0.747	96.6 (143/148)	81.6 (71/87)	15.2	<0.001
P2. We can buy antibiotic from medicine shops/pharmacies directly. (No)	41.3 (502/1216)	47.0 (285/607)	35.6 (217/609)	16.1	<0.001	52.2 (93/178)	42.9 (117/273)	3.9	0.051	49.7 (73/147)	21.8 (19/87)	17.8	<0.001
P3. We can use antibiotics after the suggestions from friends/neighbor. (No)	90.7 (1103/1216)	95.2 (577/606)	86.2 (526/610)	29.1	<0.001	94.4 (169/179)	90.9 (249/274)	2.0	0.168	98.6 (146/148)	81.6 (71/87)	22.5	<0.001
P4. Do you follow the advertisement (leaflets/internet etc.) while purchasing antibiotics? (No)	71.7 (868/1220)	75.9 (462/609)	66.4 (406/611)	13.2	<0.001	76.0 (136/179)	63.1 (173/274)	8.2	0.004	66.2 (98/148)	67.8 (59/87)	0.06	0.801
P5. If one of your family members is sick, do you share the antibiotics together? (No)	81.4 (986/121)	85.8 (521/607)	76.9 (465/605)	16.1	<0.001	81.0 (145/179)	80.8 (219/271)	0.003	0.959	87.8 (130/148)	72.4 (63/87)	8.8	0.003
P6. If you have cold and cough and the doctor does not prescribe antibiotics, what would you do? (Follow doctor’s suggestion)	90.7 (1096/1209)	94.7 (576/608)	86.5 (520/601)	24.9	<0.001	93.3 (167/179)	87.4 (236/270)	4.62	0.201	96.6 (143/148)	86.2 (75/87)	12.4	0.006
P7. Do you give the antibiotics to your friend/family if they get sick? (No)	77.7 (914/1176)	83.8 (497/593)	71.5 (417/583)	25.6	<0.001	82.8 (144/174)	79.5 (210/264)	0.69	0.403	81.4 (118/145)	57.6 (49/85)	15.2	<0.001
P8. Do you stop taking antibiotic without completing full course? (No)	85.7 (1023/1194)	92.8 (555/598)	78.5 (468/596)	49.6	<0.001	89.7 (157/175)	83.2 (223/268)	3.67	0.055	91.2 (135/148)	74.1 (106/143)	14.9	<0.001

*p* < 0.05, statistically significant.

## References

[1] Chen C., Chen Y.M., Hwang K.L. (2005). Behavior, attitudes and knowledge about antibiotic usage among residents of Changhua, Taiwan. J. Microbiol. Immunol. Infect..

[2] Levy S.B. (1998). The Challenge of Antibiotic Resistance. Sci. Am..

[3] WHO (2001). WHO Global Strategy for Containment of Antimicrobial Resistance.

[4] Okeke I.N., Laxminarayan R., Bhutta Z.A. (2005). Antimicrobial resistance in developing countries. Part I: Recent trends and current status. Lancet Infect. Dis..

[5] O’Neill J. (2014). Antimicrobial resistance: Tackling a crisis for the health and wealth of nations. Rev. Antimicrob. Resist..

[6] Okeke I.N. (2009). Poverty and root causes of resistance in developing countries. Antimicrobial Resistance in Developing Countries.

[7] WHO (2014). WHO’s First Global Report on Antibiotic Resistance Reveals Serious, Worldwide Threat to Public Health.

[8] Sawair F.A., Baqain Z.H., Karaky A.A., Eid R.A. (2009). Assessment of self-Medication of antibiotics in a jordanian population. Med. Princ. Pract..

[9] World Health Organization (2014). Antimicrobial Resistance: Global Report on Surveillance.

[10] Institute of Medicine, Board on Global Health, Forum on Emerging Infections (2003). The Resistance Phenomenon in Microbes and Infectious Disease Vectors: Implications for Human Health and Strategies for Containment Workshop Summary.

[11] Sawalha A.F. (2008). A descriptive study of self-medication practices among Palestinian medical and nonmedical university students. Res. Social Adm. Pharm..

[12] Suaifan G.A., Shehadeh M., Darwish D.A. (2012). A cross-sectional study on Knowledge, attitude and behavior related to antibiotic use and resistance among medical and non-medical university students in Jordan. Afr. J. Pharm. Pharmacol..

[13] Huang Y., Gu J., Zhang M. (2013). Knowledge, attitude and practice of antibiotics: A questionnaire study among 2500 Chinese students. BMC Med. Educ..

[14] Ganesh M., Sridevi S.A. (2014). Antibiotic use among medical and para medical students: knowledge, attitude and practice in a tertiary health care center in Chennai—A scientific insight. IJSR.

[15] Poudel A., Ibrahim M.I., Mishra P. (2017). Assessment of the availability and rationality of unregistered fixed dose drug combinations in Nepal: A multicenter cross-sectional study. Glob Health Res Policy..

[16] Basnyat B., Gelband H. (2018). Antibiotic Resistance: The growth of an unwelcome guest in Nepal. Nepali Times.

[17] Khakhurel B.K., Shakya G., Lohani G.R. (2016). National Antimicrobial Resistance Containment Action Plan Nepal.

[18] Nayak S., Rana M., Mayya S. (2016). Antibiotics to cure or harm: Concept of antibiotic resistance among health professional students in Nepal. Int. J. Med. Sci. Public Health.

[19] Sah A.K. (2016). Self-medication with antibiotics among nursing students of Nepal. IJPSR.

[20] Khan A.K.A., Banu G.K.K.R. (2013). Antibiotic Resistance and Usage—A Survey on the Knowledge, Attitude, Perceptions and Practices among the Medical Students of a Southern Indian Teaching Hospital. J. Clin. Diagn. Res..

[21] Minen M.T., Duquaine D., Marx M.A. (2010). A survey of knowledge, attitudes, and beliefs of medical students concerning antimicrobial use and resistance. Microb. Drug Resist..

[22] Rusic D., Bozic J., Vilovic M. (2018). Attitudes and Knowledge Regarding Antimicrobial Use and Resistance Among Pharmacy and Medical Students at the University of Split, Croatia. Microb. Drug Resist..

[23] Zafar S.N., Syed R., Waqar S. (2008). Self-medication amongst university students of Karachi: Prevalence, knowledge and attitudes. JPMA.

[24] Metlay J.P., Shea J.A., Crossette L.B. (2002). Tensions in antibiotic prescribing: Pitting social concerns against the interests of individual patients. J. Gen. Intern. Med..

